# Event-Triggered Tracking Control for Adaptive Anti-Disturbance Problem in Systems with Multiple Constraints and Unknown Disturbances

**DOI:** 10.3390/e25010043

**Published:** 2022-12-27

**Authors:** Hong Shen, Qin Wang, Yang Yi

**Affiliations:** 1 College of Business, Yangzhou University, Yangzhou 225127, China; 2 College of Information Engineering, Yangzhou University, Yangzhou 225127, China

**Keywords:** dynamic neural networks (DNNs), event-triggered control, anti-disturbance control, adaptive control, saturation constraint, output constraint

## Abstract

Aimed at the objective of anti-disturbance and reducing data transmission, this article discusses a novel dynamic neural network (DNN) modeling-based anti-disturbance control for a system under the framework of an event trigger. In order to describe dynamical characteristics of irregular disturbances, exogenous DNN disturbance models with different excitation functions are firstly introduced. A novel disturbance observer-based adaptive regulation (DOBAR) method is then proposed, which can capture the dynamics of unknown disturbance. By integrating the augmented triggering condition and the convex optimization method, an effective anti-disturbance controller is then found to guarantee the system stability and the convergence of the output. Meanwhile, both the augmented state and the system output are constrained within given regions. Moreover, the Zeno phenomenon existing in event-triggered mechanisms is also successfully avoided. Simulation results for the A4D aircraft models are shown to verify the availability of the algorithm.

## 1. Introduction

As is well-known, many real-world controlled systems are often subjected to unknown external disturbances [1,2,3,4,5]. Currently, there are various recognized anti-disturbance control algorithms that can be used to eliminate the effects caused by unknown disturbances, such as adaptive theory, robust control and sliding mode control [6,7,8]. However, the motivation of these methods is to suppress disturbances in the form of feedback rather than feed-forward compensation, which usually makes the reaction time linger and reduces the accuracy [1,2,9]. In order to overcome these limitations, an active feed-forward method of rejecting disturbances based on the disturbance estimation technique is proposed. This method is usually called a disturbance-observer-based control (DOBC) and can proactively offset those unknown disturbances [1,2,4,10,11,12,13,14,15,16,17]. Due to its fast reaction and good compatibility, the DOBC method has been successfully applied to many classical controlled systems, such as permanent magnet synchronous motor (PMSM) systems [11], vehicle control systems [12], Markov jump systems [13], multi-agent systems [15], non-Gaussian distribution systems [16] and so on. However, in order to better estimate disturbances, the DOBC method usually needs to acquire information on the frequency and amplitude of unknown disturbances [1,2]. As a result, most of the DOBC results can only cope with linear or regular disturbances, including constant and harmonic disturbances (see [1,2,14,15,16] for details). When being affected by those irregular nonlinear disturbances—for example, variable amplitude or frequency disturbances—how to realize the dynamic estimation is a major motivation. In short, exploring more in-depth disturbance observation strategies is one of the most important research objectives.

In either practical systems or theoretical analysis, the problem of control constraints is inevitable. As a typical input constraint phenomenon, actuator saturations frequently occur in almost all control devices and can have a great negative impact on the system performance [18]. Based on this, many researchers began to study effective saturation control algorithms [19,20,21,22,23,24,25,26,27]. In [19], multiple auxiliary matrices and convex hull partitioning methods were discussed to enlarge the ellipsoidal region of stability. By using bilinear matrix inequalities (BLMIs) or linear matrix inequalities (LMIs) schemes, the polytopic technique was explored to drag the saturation constraint into a designed convex set [20,21,22,23]. In order to obtain less conservative results, the sector bounding approach also became popular for describing the saturation function [22]. Moreover, when coupling with other nonlinear characteristics or typical controlled systems, corresponding anti-windup strategies and performance analysis were also discussed in [22,23,24,25,26,27]. Parallel to the input constraint, both the output and state-constrained controls are also attractive topics driven by both practical and theoretical requirements [28,29]. Among the existing results, the symmetric barrier Lyapunov function (BLF), asymmetric BLF and error transformation proved to be effective in dealing with output constraints [28,29,30,31]. However, the aforementioned discussions are only limited to the single-input single-output (SISO) systems or triangular multiple-input multiple-output (MIMO) systems. It is urgent to explore new control methods to guarantee the state or output constraints of general MIMO nonlinear systems. Further, when multiple constraints and unknown disturbances are coupled, how to design an effective anti-disturbance constrained controller is another motivation of the work.

Generally, most controlled systems adopt a time-triggered mechanism (also called periodic sampling mechanism), which is rather convenient for theoretical analysis and conventional engineering applications. However, when the system performance has reached the designed requirements in networked environments, data transmission and calculation do not stop immediately, which will inevitably cause a waste of bandwidths and computing resources to a certain extent [32]. Due to this consideration, the idea of event triggering is proposed by equipping event-triggered schedulers at sensor nodes [33,34]. In the event-triggered control (ETC) framework, control tasks are carried out only after the well-designed triggering criteria are violated, which can availably decrease resource utilization while achieving a satisfactory system performance [35]. Some exciting results regarding ETC systems have successfully addressed traditional problems of robust control, output feedback control, sliding mode control, adaptive control, and so on [34,35,36,37,38,39,40]. In practical applications, Ref. [41] proposed an effective decentralized event-triggered algorithm to guarantee the dynamical performance of power systems. Based on the event-triggered theory, the effective attitude tracking control was discussed for the surface vessels [42].

On the basis of the analysis above, this paper explores a novel event-based anti-disturbance constraint control problem for general MIMO systems subject to unknown disturbances and multiple constraints. The proposed scheme has the following characteristics. Firstly, a DNN disturbance model was employed to identify those indescribable irregular disturbances, which further enriches the varieties of disturbances when compared with most existing anti-disturbance results [1,2,11,13,14,15]. By designing the adaptive law for adjustable parameters of DNNs, an active disturbance-observer-based adaptive control (DOBAC) algorithm was designed to successfully realize the dynamical estimation and rejection of unknown disturbances. Secondly, in order to avoid the waste of resources and achieve favorable dynamical tracking, an event-triggered mechanism with the designed augmented triggering condition was introduced into the controlled system. Further, a composite event-triggered anti-disturbance controller can be smoothly implemented after decoupling the saturated input with the disturbances. Thirdly, unlike many previous non-convex results [20,22], the improved convex optimization algorithm was constructed to simultaneously satisfy the multi-objective control requirements, including the stability of the augmented system, dynamical tracking performance, state constraint, output constraint and non-Zeno phenomenon. It also represents a major expansion with respect to those single-constraint control or dynamical tracking problems. By introducing two kinds of different disturbances, the simulation examples of the A4D model are presented to reflect the significance of the algorithm.

## 2. Problem Description

Considering the MIMO system with external disturbances and an input constraint as

(1) $$\left\{\begin{array}{cc} \dot{x}\left(t\right) & =Ax(t)+B\mathrm{sat}(u(t)+g(t))\\ z(t) & =Cx(t) \end{array}\right.$$

where $u\left(t\right)\in R^{m}$, $z\left(t\right)\in R^{p}$, $x\left(t\right)\in R^{n}$ and $g\left(t\right)\in R^{m}$ are, respectively, the control input, the system output, the state vector and the unknown disturbance. $A\in R^{n\times n}$, $B\in R^{n\times m}$ and $C\in R^{p\times n}$ are the coefficient matrices. sat(∗) stands for the saturation constraint, which is expanded as $\mathrm{sat}\left(*\right)={\left[sat_{1}\left(*\right),\ldots ,sat_{m}\left(*\right)\right]}^{T}$, where $sat_{i}\left(*\right)=sign\left(*\right)\min\left(*,1\right)$ stands for the signum function.

To better estimate unknown disturbances, g(t) is described by an external model with adjustable parameters as

(2) $$\left\{\begin{array}{cc} \dot{\sigma }\left(t\right) & =W\sigma \left(t\right)+M^{*}\Phi \left(\sigma \left(t\right)\right)\\ g(t) & =V\sigma (t) \end{array}\right.$$

where $\sigma \left(t\right)\in R^{n_{1}}$ represents the middle state of the DNN model, and *W* and *V* are corresponding coefficient matrices. In addition, $M^{*}\in R^{n_{1}\times n_{1}}$ represents the optimal model parameter matrix, and Φ(∗) can be seen as the activation function of DNNs with $\Phi \left(*\right)={\left[\phi_{1}\left(*\right),\ldots ,\phi_{n_{1}}\left(*\right)\right]}^{T}$. Due to the powerful identification capacity of DNNs (see [43,44]), DNN models ought to be useful identifiers to depict different types of disturbances by selecting different activation functions.

For the purpose of achieving a favorable dynamic tracking performance, an augmented state is defined as

(3) $$\bar{x}\left(t\right)={\left[x^{T}\left(t\right),\int_{0}^{t}e^{T}\left(\tau \right)d\tau \right]}^{T}$$

where the error is defined by $e\left(t\right):=z\left(t\right)-z_{d}$ with $z_{d}$ standing for the expected system output, and $z_{d}$ is a nonzero vector. According to (1) as well as (3), the extended system can be expressed by

(4) $$\left\{\begin{array}{cc} \dot{\bar{x}}\left(t\right) & =\bar{A}\bar{x}\left(t\right)+\bar{B}\mathrm{sat}\left(u\left(t\right)+g\left(t\right)\right)+\bar{G}z_{d}\\ z(t) & =\bar{C}\bar{x}\left(t\right) \end{array}\right.$$

with

$$\bar{A}=\left[\begin{array}{cc} A & 0\\ C & 0 \end{array}\right],\;\;\bar{B}=\left[\begin{array}{c} B\\ 0 \end{array}\right],\;\;\bar{G}=\left[\begin{array}{c} 0\\ -I \end{array}\right],\;\;\bar{C}={\left[\begin{array}{c} C^{T}\\ 0 \end{array}\right]}^{T}$$

Moreover, the polyhedron boundary skill is employed to identify the function with saturation. By selecting a matrix $P_{1}$, the ellipsoid is constructed as

(5) $$\Lambda \left(P_{1},1\right)=\left\{\bar{x}\left(t\right)\in R^{n+p}:{\bar{x}}^{T}P_{1}\bar{x}\leq 1\right\}$$

Based on this, a polyhedron is structured as

(6) $$L\left(H\right)=\left\{\bar{x}\left(t\right)\in R^{n+p}:\left|H^{l}\bar{x}\right|\leq 1,l\in Q_{m}\right\}$$

where $Q_{m}=\left\{1,2,\cdot \cdot \cdot ,m\right\}$,$H^{l}$ stands for the *l*th row of *H*. Further, the lemma is imported.

**Lemma 1**  ([18,19,20]). *Let $K,H\in R^{m\times (n+p)}$. For every $\zeta \in R^{n+p}$, if ζ∈L(H), then*

(7) $$\mathrm{sat}\left(K\zeta \right)=co\left\{D_{i}K\zeta +D_{i}^{-}H\zeta ,i\in Q\right\}$$

*where co(∗) stands for the convex hull representation, and $Q=\left\{1,\cdots ,2^{m}\right\}$. In addition, $D_{i}$ is a diagonal matrix, in which each element is 0 or 1, and it satisfies $D_{i}+D_{i}^{-}=I$.*

## 3. Event-Triggered PI Controller Design

For reducing the waste of resources in networked environments, an event-trigger-based proportional-integral (PI) controller is designed in this part.

First, the novel augmented event triggering condition is defined as

(8) $$\begin{array}{c} t_{k+1}=\inf\left\{t>t_{k}:{\left(\bar{x}\left(t\right)-\bar{x}\left(t_{k}\right)\right)}^{T}\Psi \left(\bar{x}\left(t\right)-\bar{x}\left(t_{k}\right)\right)>\delta^{2}{\bar{x}}^{T}\left(t\right)\Psi \bar{x}\left(t\right)\right\} \end{array}$$

where $t_{k}$ represents the moment at which the event is triggered in *k*th, and $\bar{x}\left(t\right)$ and $\bar{x}\left(t_{k}\right)$ are the augmented states at the current sampling time and the latest triggered time. The scalar δ satisfies 0≤δ<1, and Ψ>0 represents a designed positive definite matrix.

Define

(9) $$e_{k}\left(t\right)=\left[\begin{array}{c} e_{1k}\\ e_{2k} \end{array}\right],\;\;t\in \left[t_{k},t_{k+1}\right)$$

with

(10) $$\left\{\begin{array}{cc} e_{1k}\left(t\right) & =x\left(t\right)-x\left(t_{k}\right)\\ e_{2k}\left(t\right) & =\int_{0}^{t}e\left(\tau \right)d\tau -\int_{0}^{t_{k}}e\left(\tau \right)d\tau =\int_{t_{k}}^{t}e\left(\tau \right)d\tau \end{array}\right.$$

Then, we can derive from (9) and (10) that

(11) $${\dot{e}}_{k}\left(t\right)=\left[\begin{array}{c} \dot{x}\left(t\right)\\ e(t) \end{array}\right]=\left[\begin{array}{c} \dot{x}\left(t\right)\\ Cx\left(t\right)-z_{d} \end{array}\right]$$

In the event-triggered mechanism, the event trigger monitors whether the events occur. Once the triggering condition $e_{k}^{T}\left(t\right)\Psi e_{k}\left(t\right)\leq \delta^{2}{\bar{x}}^{T}\left(t\right)\Psi \bar{x}\left(t\right)+\delta_{1}e^{-\varsigma t}$ is not met, a new event will occur. The event detector then sends the updated data $\bar{x}\left(t\right)$ to the control port. Otherwise, the current updated data will be put away.

Based on this, the event-triggered PI state feedback controller is expressed by the form

(12) $$u\left(t\right)=-\hat{g}\left(t\right)+K\bar{x}\left(t_{k}\right),\;K=\left[K_{P},K_{I}\right],\;t\in \left[t_{k},t_{k+1}\right)$$

where $K_{P}$ and $K_{I}$ stand for the control gains to be sought.

## 4. Event-Triggered DOBAC Algorithm Design

For the sake of estimating unknown disturbance g(t) accurately, an adaptive observer with adjustable weight is built. The specific expression of the adaptive DO is described as

(13) $$\left\{\begin{array}{cc} \dot{r}\left(t\right) & =\hat{M}\left(t\right)\Phi \left(\hat{\sigma }\left(t\right)\right)-L\left(-\bar{A}\bar{x}\left(t\right)-\bar{G}z_{d}-\bar{B}u\left(t\right)\right)+\left(W\;+\;L\bar{B}V\right)\left(-L\bar{x}\left(t\right)+r\left(t\right)\right)\\ \hat{\sigma }\left(t\right) & =-L\bar{x}\left(t\right)+r\left(t\right)\\ \hat{g}\left(t\right) & =V\hat{\sigma }\left(t\right) \end{array}\right.$$

where *L* is the gain to be devised later, r(t) represents the instrumental variable and $\hat{M}\left(t\right)$ is the adjustable dynamical weight, and its adaptive law is defined as

(14) $$\dot{\hat{M}}\left(t\right)=-∥\hat{\sigma }\left(t\right)∥\hat{M}\left(t\right)+\gamma P_{2}\hat{\sigma }\left(t\right)\Phi^{T}\left(\hat{\sigma }\left(t\right)\right)$$

where γ>0 is a given parameter and $P_{2}>0$ will be solved in the next section.

The following theorem gives the boundedness proof of the adjustable parameter $\hat{M}\left(t\right)$.

**Theorem 1.**  *If the adaptive parameter $\hat{M}\left(t\right)$ is updated by (14) and the initial condition satisfies $\hat{M}\left(0\right)\in \Theta_{\hat{M}}$, then $\hat{M}\left(t\right)\in \Theta_{\hat{M}}$ will be guaranteed for all t≥0, where*

$$\Theta_{\hat{M}}=\left\{\hat{M}\left(t\right)|∥\hat{M}{∥}_{F}\leq \gamma \sqrt{n_{1}}∥P_{2}∥\right\}$$

*is a known compact set.*

**Proof.**  Design the function as

(15) $$\Gamma \left(t\right)=\frac{1}{2}tr\left\{{\hat{M}}^{T}\left(t\right)\gamma^{-1}\hat{M}\left(t\right)\right\}.$$

According to the above Formula (14), we have

(16) $$\begin{array}{c} \dot{\Gamma }=-\gamma^{-1}∥\hat{\sigma }\left(t\right)∥∥\hat{M}{\left(t\right)∥}_{F}^{2}+∥{\hat{\sigma }}^{T}\left(t\right)P_{2}\hat{M}\left(t\right)\Phi \left(\hat{\sigma }\left(t\right)\right)∥ \end{array}$$

The excitation function is chosen as

$$\Phi \left(\sigma \left(t\right)\right)={\left[1/\left(e^{-\kappa \sigma_{1}}+1\right),\cdots ,1/\left(e^{-\kappa \sigma_{n_{1}-1}}+1\right),1\right]}^{T}$$

where κ is a positive constant. The boundary condition $∥\Phi \left(\hat{\sigma }\left(t\right)\right)∥\leq \sqrt{n_{1}}$ can easily be achieved. Further, (16) is rewritten as

(17) $$\begin{array}{cc} \dot{\Gamma } & =∥\hat{\sigma }\left(t\right)∥∥\hat{M}{∥}_{F}\left(\gamma^{-1}∥\hat{M}{∥}_{F}-\sqrt{n_{1}}∥P_{2}∥\right) \end{array}$$

which certifies that $\dot{\Gamma }\left(t\right)\leq 0$ once the inequality $∥\hat{M}{\left(t\right)∥}_{F}>\gamma \sqrt{n_{1}}∥P_{2}∥$ holds. Hence, if the initial condition satisfies $\hat{M}\left(0\right)\in \Theta_{\hat{M}}$, then holds $\hat{M}\left(t\right)\in \Theta_{\hat{M}}$ holds. □

The following discussion is concerned with the decoupling problem of a nonlinear saturated input under the event-triggered framework. According to Lemma 1, by choosing $H=[H_{1},-V]$ to satisfy η(t)∈L(H), $\forall t\in [t_{k},t_{k+1})$, one has

(18) $$\begin{array}{c} \mathrm{sat}\left(u\left(t\right)+g\left(t\right)\right)=\sum_{i=1}^{2^{m}}\chi_{i}\left(D_{i}K+D_{i}^{-}H_{1}\right)\bar{x}\left(t_{k}\right)-Ve_{\sigma }\left(t\right) \end{array}$$

where the scalars $\chi_{i}$ meet the condition $0\leq \chi_{i}\leq 1$ and $\sum_{i=1}^{2^{m}}\chi_{i}=1$. $e_{\sigma }\left(t\right)=\hat{\sigma }\left(t\right)-\sigma \left(t\right)$, $\eta \left(t\right)={\left[{\bar{x}}^{T}\left(t_{k}\right),e_{\sigma }^{T}\left(t\right)\right]}^{T}$.

Introducing the input (18) to the system (4) results in the form

(19) $$\begin{array}{c} \dot{\bar{x}}\left(t\right)=\left(\bar{A}+\sum_{i=1}^{2^{m}}\chi_{i}\bar{B}\left(D_{i}K+D_{i}^{-}H_{1}\right)\right)\bar{x}\left(t\right)-\sum_{i=1}^{2^{m}}\chi_{i}\bar{B}\left(D_{i}K+D_{i}^{-}H_{1}\right)e_{k}\left(t\right)-\bar{B}Ve_{\sigma }\left(t\right)+\bar{G}y_{d} \end{array}$$

Defining $\tilde{M}\left(t\right)=M^{*}-\hat{M}\left(t\right)$ and applying (2), (13) and (17), we arrive at

(20) $$\begin{array}{cc} {\dot{e}}_{\sigma }\left(t\right) & =\left(W+L\bar{B}V\right)e_{\sigma }\left(t\right)-\sum_{i=1}^{2^{m}}\chi_{i}L\bar{B}D_{i}^{-}\left(H_{1}-K\right)\bar{x}\left(t\right)-\tilde{M}\left(t\right)\Phi \left(\hat{\sigma }\left(t\right)\right)\\ \; & +\sum_{i=1}^{2^{m}}\chi_{i}L\bar{B}D_{i}^{-}\left(H_{1}-K\right)e_{k}\left(t\right)+M^{*}\left(\Phi \left(\hat{\sigma }\left(t\right)\right)-\Phi \left(\sigma \left(t\right)\right)\right) \end{array}$$

Further, by integrating the system (19) with the error dynamic system (20), we can obtain

(21) $$\begin{array}{c} \dot{\xi }\left(t\right)=\tilde{A}\xi \left(t\right)+\tilde{G}y_{d}+\tilde{I}\left(M^{*}\left(\Phi \left(\hat{\sigma }\left(t\right)\right)-\Phi \left(\sigma \left(t\right)\right)\right)-\tilde{M}\Phi \left(\hat{\sigma }\left(t\right)\right)\right) \end{array}$$

where

$$\xi \left(t\right)=\left[\begin{array}{c} \bar{x}\left(t\right)\\ e_{\sigma }\left(t\right)\\ e_{k}\left(t\right) \end{array}\right],\;\;\tilde{G}=\left[\begin{array}{c} \bar{G}\\ 0\\ \bar{G} \end{array}\right],\;\;\tilde{I}=\left[\begin{array}{c} 0\\ I\\ 0 \end{array}\right],\;\;\tilde{A}=\left[\begin{array}{ccc} \bar{A}+\prod_{11} & -\bar{B}V & -\prod_{11}\\ -\prod_{21} & W+L\bar{B}V & \prod_{21}\\ \bar{A}+\prod_{11} & -\bar{B}V & -\prod_{11} \end{array}\right]$$

$$\prod_{11}=\sum_{i=1}^{2^{m}}\chi_{i}\bar{B}\left(D_{i}K+D_{i}^{-}H_{1}\right),\;\;\prod_{21}=\sum_{i=1}^{2^{m}}\chi_{i}L\bar{B}D_{i}^{-}\left(H_{1}-K\right)$$

In the next section, by importing the convex optimization method, the desirable gains *K* and *L* will be given to meet the multi-objective control requirements of the augmented system.

## 5. Analysis and Proof of Multi-Objective Tracking Control Performance

For the sake of ensuring the performance of the closed-loop system, some related assumptions are necessary.

**Assumption A1.**  *The selected basis function Φ(∗) is assumed to satisfy the following Lipschitz condition:*

(22) $${\left(\Phi \left(\sigma \right)-\Phi \left(\hat{\sigma }\right)\right)}^{T}\left(\Phi \left(\sigma \right)-\Phi \left(\hat{\sigma }\right)\right)\leq e_{\sigma }^{T}\left(t\right)U_{\sigma }^{T}U_{\sigma }e_{\sigma }\left(t\right)$$

*where $U_{\sigma }$ is a known positive definite matrix.*

**Assumption A2.**  *The optimal parameter $M^{*}$ is usually an unknown bounded matrix, so there exists a positive definite matrix $\bar{M}$ satisfying the inequality $M^{*T}M^{*}\leq \bar{M}$.*

**Assumption A3.**  *The unknown disturbance g(t) is supposed to satisfy the condition $g^{T}\left(t\right)g\left(t\right)\leq \theta_{g}$, where $\theta_{g}$ is a constant. Further, because of $g^{T}\left(t\right)g\left(t\right)=\sigma^{T}\left(t\right)V^{T}V\sigma \left(t\right)\leq \theta_{g}$, another inequality condition follows: $\sigma^{T}\left(t\right)\sigma \left(t\right)\leq \frac{\theta_{g}}{\lambda_{\min}\left(V^{T}V\right)}$.*

In this section, the following four theorems will give the relevant proofs of dynamic performances of the closed-loop system (19), including the stability, dynamical tracking, output constraint and non-Zeno phenomenon.

**Theorem 2.**  *For given parameters $\mu_{i}>0,i=1,2$, δ>0 and $\delta_{1}>0$, if there exist the matrices $\tilde{\Psi }>0$, $Q_{1}=P_{1}^{-1}>0$, $P_{2}>0$ and $R_{i},i=1,2,3$, the following inequality is made:*

(23) $$\left[\begin{array}{ccccc} \sigma_{11} & \sigma_{12} & \sigma_{13} & \bar{G} & 0\\ {}* & \sigma_{22} & \sigma_{23} & 0 & P_{2}\\ {}* & * & \mu^{2}\Psi^{-1}-2\mu Q_{1} & 0 & 0\\ {}* & * & * & -\mu_{1}^{2}I & 0\\ {}* & * & * & * & -{\bar{M}}^{-1} \end{array}\right]<0$$

*where*

$$\left\{\begin{array}{cc} \sigma_{11} & =sym\left\{\bar{A}Q_{1}+\sum_{i=1}^{2^{m}}\chi_{i}\bar{B}\left(D_{i}R_{1}+D_{i}^{-}R_{2}\right)\right\}+\delta^{2}\tilde{\Psi }+Q_{1}\\ \sigma_{12} & =-\bar{B}V-{\left(\sum_{i=1}^{2^{m}}\chi_{i}R_{3}\bar{B}D_{i}^{-}\left(R_{2}-R_{1}\right)\right)}^{T}\\ \sigma_{13} & =-\sum_{i=1}^{2^{m}}\chi_{i}\bar{B}\left(D_{i}R_{1}+D_{i}^{-}R_{2}\right)\\ \sigma_{22} & =sym\left\{P_{2}W+R_{3}\bar{B}V\right\}+U_{\sigma }^{T}U_{\sigma }+\mu_{2}^{-2}I+P_{2}\\ \sigma_{23} & =\sum_{i=1}^{2^{m}}\chi_{i}R_{3}\bar{B}D_{i}^{-}\left(R_{2}-R_{1}\right) \end{array}\right.$$

*is solvable, and the adaptive law of $\hat{M}\left(t\right)$ is designed by (14); then, both the controlled system (19) and the dynamical error system (20) will be stable and the augmented variable ξ(t) will retain a small set $\Theta_{\xi (t)}$, where*

$$\Theta_{\xi (t)}=\left\{\xi \left(t\right)|∥\xi \left(t\right)∥\leq \sqrt{\left(\mu_{1}^{2}y_{d}^{2}+\kappa \right)/\lambda_{\min}\left(P_{1}\right)}\right\}.$$

*Moreover, the gain matrices K, $H_{1}$, L and *Ψ* are, respectively, given by*

$$K=R_{1}Q_{1}^{-1},\;H_{1}=R_{2}Q_{1}^{-1},\;L=P_{2}^{-1}R_{3},\;\tilde{\Psi }=Q_{1}\Psi Q_{1}$$

**Proof.**  Select the Lyapunov functions as

(24) $$V_{1}\left(\bar{x}\left(t\right),t\right)={\bar{x}}^{T}\left(t\right)P_{1}\bar{x}\left(t\right)$$

and

(25) $$V_{2}\left(e_{\sigma }\left(t\right),t\right)=e_{\sigma }^{T}\left(t\right)P_{2}e_{\sigma }\left(t\right)+tr\left\{{\tilde{M}}^{T}\left(t\right)\gamma^{-1}\tilde{M}\left(t\right)\right\}$$

Along the trajectory of (19), we have, from (24), that

(26) $$\begin{array}{cc} \dot{V_{1}} & \leq {\bar{x}}^{T}\left(t\right)sym\left\{P_{1}\bar{A}+\sum_{i=1}^{2^{m}}\chi_{i}P_{1}\bar{B}\left(D_{i}K+D_{i}^{-}H_{1}\right)\right\}\bar{x}\left(t\right)+{\bar{x}}^{T}\left(t\right)\left\{\mu_{1}^{-2}P_{1}\bar{G}{\bar{G}}^{T}P_{1}+\delta^{2}\Psi \right\}\bar{x}\left(t\right)\\ \; & -2{\bar{x}}^{T}\left(t\right)\sum_{i=1}^{2^{m}}\chi_{i}P_{1}\bar{B}\left(D_{i}K+D_{i}^{-}H_{1}\right)e_{k}\left(t\right)-2{\bar{x}}^{T}P_{1}\bar{B}Ve_{\sigma }\left(t\right)+\mu_{1}^{2}z_{d}^{2}-e_{k}^{T}\left(t\right)\Psi e_{k}\left(t\right) \end{array}$$

The derivative of $V_{2}$ along (20) is deduced by

(27) $$\begin{array}{cc} \dot{V_{2}} & \leq e_{\sigma }^{T}\left(t\right)\left(sym\left(P_{2}W\;+\;P_{2}L\bar{B}V\right)\;+\;P_{2}\bar{M}P_{2}\;+\;U_{\sigma }^{T}U_{\sigma }\right)e_{\sigma }\left(t\right)\\ \; & -2e_{\sigma }^{T}\left(t\right)\sum_{i=1}^{2^{m}}\chi_{i}P_{2}L\bar{B}D_{i}^{-}\left(H_{1}-K\right)\bar{x}\left(t\right)+2e_{\sigma }^{T}\left(t\right)\sum_{i=1}^{2^{m}}\chi_{i}P_{2}L\bar{B}D_{i}^{-}\left(H_{1}-K\right){\bar{e}}_{k}\left(t\right)\\ \; & +2∥\hat{\sigma }\left(t\right)∥∥M^{*}{∥}_{F}^{2}+\sqrt{\frac{2\theta_{g}n_{1}}{\lambda_{\min}\left(V^{T}V\right)}}∥P_{2}∥\left(\gamma \sqrt{n_{1}}P_{2}+\sqrt{tr(\bar{M})}\right) \end{array}$$

Notice that

(28) $$\begin{array}{cc} 2∥\hat{\sigma }\left(t\right)∥∥M^{*}{∥}_{F}^{2} & \leq 2∥\sigma \left(t\right)∥∥M^{*}{∥}_{F}^{2}+2∥e_{\sigma }\left(t\right)∥∥M^{*}{∥}_{F}^{2}\\ \; & \leq 2\sqrt{\frac{\theta_{g}}{\lambda_{\min}\left(V^{T}V\right)}}tr\left(\bar{M}\right)+\mu_{2}^{2}{\left(tr(\bar{M})\right)}^{2}+\mu_{2}^{-2}e_{\sigma }^{T}\left(t\right)e_{\sigma }\left(t\right) \end{array}$$

Then, integrating (26) and (27) with (28) produces

(29) $$\dot{V_{1}}+\dot{V_{2}}\leq \xi^{T}\left(t\right)\Omega \xi \left(t\right)+\mu_{1}^{2}y_{d}^{2}+\kappa$$

where the parameter κ is expressed as

(30) $$\begin{array}{c} \kappa =\sqrt{\frac{2\theta_{g}n_{1}}{\lambda_{\min}\left(V^{T}V\right)}}∥P_{2}∥\left(\gamma \sqrt{n_{1}}P_{2}+\sqrt{tr(\bar{M})}\right)+2\sqrt{\frac{\theta_{g}}{\lambda_{\min}\left(V^{T}V\right)}}tr\left(\bar{M}\right)+\mu_{2}^{2}{\left(tr(\bar{M})\right)}^{2} \end{array}$$

and

(31) $$\Omega =\left[\begin{array}{ccc} \varpi_{11} & \varpi_{12} & \varpi_{13}\\ {}* & \varpi_{22} & \varpi_{23}\\ {}* & * & -\Psi \end{array}\right]$$

with

$$\left\{\begin{array}{cc} \varpi_{11} & =sym\left\{P_{1}\bar{A}+P_{1}\sum_{i=1}^{2^{m}}\chi_{i}\bar{B}\left(D_{i}K+D_{i}^{-}H_{1}\right)\right\}+\mu_{1}^{-2}P_{1}\bar{G}{\bar{G}}^{T}P_{1}+\delta^{2}\Psi \\ \varpi_{12} & =-P_{1}\bar{B}V-{\left(P_{2}\sum_{i=1}^{2^{m}}\chi_{i}L\bar{B}D_{i}^{-}\left(H_{1}-K\right)\right)}^{T}\\ \varpi_{13} & =-P_{1}\sum_{i=1}^{2^{m}}\chi_{i}\bar{B}\left(D_{i}K+D_{i}^{-}H_{1}\right)\\ \varpi_{22} & =sym\left\{P_{2}\left(W+L\bar{B}V\right)\right\}+P_{2}\bar{M}P_{2}+U_{\sigma }^{T}U_{\sigma }+\mu_{2}^{-2}I\\ \varpi_{23} & =P_{2}\sum_{i=1}^{2^{m}}\chi_{i}L\bar{B}D_{i}^{-}\left(H_{1}-K\right). \end{array}\right.$$

Based on the Lemma 2, by multiplying the matrix $diag\left\{P_{1},I,P_{1},I,I,I\right\}$ to two sides of (23), we have

(23) ⟺ Ω < *diag*{*P*_1_, *P*_2_, 0}

Then, (29) is expressible as

(32) $${\dot{V}}_{1}+{\dot{V}}_{2}\leq -\xi^{T}\left(t\right)\tilde{P}\xi \left(t\right)+\mu_{1}^{2}z_{d}^{2}+\kappa$$

where $\tilde{P}=diag\left\{P_{1},P_{2},\alpha I\right\}$, with α being a proper positive constant. If

$$\xi^{T}\left(t\right)\tilde{P}\xi \left(t\right)>\mu_{1}^{2}z_{d}^{2}+\kappa$$

then it is easy to arrive at

$$\dot{V_{1}}+\dot{V_{2}}<0.$$

Thus, for any $\bar{x}\left(t\right)$, $e_{\sigma }\left(t\right)$ and $e_{k}\left(t\right)$, we have

(33) $$\xi^{T}\left(t\right)\tilde{P}\xi \left(t\right)\leq \max\left\{\xi^{T}\left(0\right)\tilde{P}\xi \left(0\right),\mu_{1}^{2}z_{d}^{2}+\kappa \right\}=\pi$$

which implies that the controlled system (21) is stable with the original state ξ(0). Thus, the state ξ(t) can be ensured to converge into $\Theta_{\xi (t)}$. The proof is complete. □

**Theorem 3.**  *For given positive parameters $\mu_{i},i=1,2$ and δ, if there exists $P_{1}^{-1}=Q_{1}>0$, $P_{2}>0$, $\tilde{\Psi }>0$ and $R_{i},i=1,2,3$ satisfying (23) and the conditions*

(34) $$\begin{array}{cc} \; & \left[\begin{array}{cc} Q_{1} & Q_{1}{\bar{C}}_{i}^{T}\\ {}* & \left(\pi^{-1}z_{di}^{2}\right)I \end{array}\right]\geq 0,\;i=1,2,\cdots ,p \end{array}$$

(35) $$\begin{array}{cc} \; & \left[\begin{array}{ccc} \pi^{-1} & R_{2}^{l} & V^{l}\\ {}* & Q_{1} & 0\\ {}* & * & P_{2} \end{array}\right]\geq 0,l=1,2,\cdots ,m \end{array}$$

*where ${\bar{C}}_{i}$ and $z_{di}$, respectively, represent the ith row of $\bar{C}$ and the ith component of $z_{d}$, $R_{2}^{l}$ and $V^{l}$ are, respectively, the ith row of $R_{2}$ and V and the adaptive regulation law of $\hat{M}\left(t\right)$ is designed by (14), the augmented system (21) will be stable and the tracking error of the output will astringe to zero; that is,*

$$\lim_{t\to \infty }z\left(t\right)=z_{d}$$

*Moreover, the state saturation constraint η(t)∈L(H) will also be satisfied. In addition, the gain matrices K, $H_{1}$, L and *Ψ* are, respectively, given by*

$$K=R_{1}Q_{1}^{-1},\;H_{1}=R_{2}Q_{1}^{-1},\;L=P_{2}^{-1}R_{3},\;\tilde{\Psi }=Q_{1}\Psi Q_{1}$$

**Proof.**  Similar to the above Theorem, the stability of the augmented system (21) will be proved. From (34), it is not hard to deduce that

$${\bar{C}}_{i}^{T}{\bar{C}}_{i}\leq \pi^{-1}z_{di}^{2}P_{1}.$$

Thus, the inequality can be obtained by

(36) $$\begin{array}{c} z_{i}^{2}\left(t\right)={\bar{x}}^{T}\left(t\right){\bar{C}}_{i}^{T}{\bar{C}}_{i}\bar{x}\left(t\right)\leq \pi^{-1}z_{di}^{2}{\bar{x}}^{T}\left(t\right)P_{1}\bar{x}\left(t\right)\leq z_{di}^{2} \end{array}$$

On one hand, it can be known that the term $\int_{0}^{t}e\left(\tau \right)d\tau$ is a part of $\bar{x}\left(t\right)$. Therefore, when t→+∞, it can be verified that the integral item must be bounded. Meanwhile, due to the constraint condition of each component of the output (36), the sign of e(t) will stay the same for all t≥0. In general, it can be concluded that the tracking error satisfies $\lim_{t\to \infty }z\left(t\right)=z_{d}$.

On the other hand, according to the Theorem 1, the η(t) will stay in the defined ellipsoid $\Omega \left(\bar{P},\pi \right)$, where $\bar{P}=diag\left\{P_{1},P_{2}\right\}$. In addition, by multiplying left and right sides of (35) with the matrix $diag\{I,Q_{1}^{-1},I\}$, one has

(37) $$\left[\begin{array}{ccc} \pi^{-1} & H_{1}^{l} & V^{l}\\ {}* & P_{1} & 0\\ {}* & * & P_{2} \end{array}\right]>0$$

Applying the Schur formula into (37) yields

(38) $${\left(H^{l}\eta \left(t\right)\right)}^{T}\left(H^{l}\eta \left(t\right)\right)\leq \pi^{-1}\eta^{T}\left(t\right)\bar{P}\eta \left(t\right)\leq 1$$

Thus, it can be inferred that $\Omega \left(\bar{P},\pi \right)\subset L\left(H\right)$ can be met for all η(t). Therefore, η(t)∈L(H) can be pledged for all $\eta \left(t\right)\in \Omega \left(\bar{P},\pi \right)$. □

The next theorem is concerned with the problem of how to determine the minimum triggering time interval.

**Theorem 4.**  *For the system (4), under the designed event-triggering format (8), the minimum triggering interval can be given by*

(39) $$\tilde{T}=\min_{k}\left\{t_{k+1}-t_{k}\right\}=\frac{1}{a}\ln\left(1+\frac{a}{b}\Delta \left(t\right)\right)>0$$

*where*

(40) $$\begin{array}{c} a=\left|\lambda_{\max}\left(\bar{A}\right)\right|,\;\;b=a∥\bar{B}∥∥\bar{x}\left(t_{k}\right)∥+∥\bar{G}∥∥z_{d}∥,\;\;\Delta \left(t\right)=\delta \frac{\lambda_{\max}\left(\Psi \right)}{\lambda_{\min}\left(\Psi \right)}∥\bar{x}\left(t\right)∥ \end{array}$$

**Proof.**  From (9), it is obtained that

$${\dot{e}}_{k}\left(t\right)=\bar{A}\bar{x}\left(t\right)+\bar{B}sat\left(u\left(t\right)+g\left(t\right)\right)+\bar{G}z_{d}$$

Furthermore, for all $t\in [t_{k},t_{k+1})$, one has

(41) $$\begin{array}{c} \frac{d}{dt}∥e_{k}\left(t\right)∥\leq |\lambda_{\max}\left(\bar{A}\right)|∥e_{k}\left(t\right)∥+|\lambda_{\max}\left(\bar{A}\right)|∥\bar{x}\left(t_{k}\right)∥+∥\bar{B}∥+∥\bar{G}∥∥z_{d}∥ \end{array}$$

By defining *a* and *b* as given in (40), the inequality (41) is described as

(42) $$\frac{d}{dt}∥e_{k}\left(t\right)∥\leq a∥e_{k}\left(t\right)∥+b$$

It is easy to deduce that

$$∥\Psi^{\frac{1}{2}}e_{k}\left(t\right)∥\leq \frac{a}{b}\left(e^{a(t-t_{k})}-1\right)$$

Based on the event-triggering condition, by solving $\Delta \left(t\right)=\frac{a}{b}\left(e^{a(t-t_{k})}-1\right)$, we can achieve

$$\tilde{T}=\frac{1}{a}\ln\left(1+\frac{a}{b}\Delta \left(t\right)\right)$$

which is the minimum triggering time interval. Based on the definition of $\bar{x}\left(t\right)$, ∥x(t)∥≠0 is true. Thus, the minimum triggering time interval $\tilde{T}>0$ holds. In conclusion, the Zeno phenomenon will not happen in the designed event-triggered algorithm. □

Please note that (23) in Theorem 2 is not a standard LMI and is actually a BLMI. Generally, the BLMI can be solved by fixing the matrix $R_{3}$ or the matrices $R_{1}$ and $R_{2}$ beforehand. As such, the results in Theorem 2 really do not give a convex optimization algorithm. Therefore, the next theorem intends to further improve the results of Theorem 2.

**Theorem 5.**  *Given parameters $\mu_{i}>0$, $\alpha_{i}>0$, δ>0 and $\delta_{1}>0$, if there are matrices $P_{2}>0$, $Q_{1}=P_{1}^{-1}>0$, $\tilde{\Psi }>0$, R>0 and $R_{i}$ such that the conditions*

(43) $$\left[\begin{array}{ccccccc} \psi_{11} & \psi_{12} & \psi_{13} & \bar{G} & 0 & Q_{1} & 0\\ {}* & \psi_{22} & 0 & 0 & P_{2} & 0 & 0\\ {}* & * & \psi_{33} & 0 & 0 & 0 & Q_{1}\\ {}* & * & * & -\mu_{1}^{2}I & 0 & 0 & 0\\ {}* & * & * & * & -{\bar{M}}^{-1} & 0 & 0\\ {}* & * & * & * & * & -\alpha_{1}^{-1}I & 0\\ {}* & * & * & * & * & * & -\alpha_{3}^{-1}I \end{array}\right]<0$$

*with*

$$\left\{\begin{array}{cc} \psi_{11} & =sym\left\{\bar{A}Q_{1}+\sum_{i=1}^{2^{m}}\chi_{i}\bar{B}\left(D_{i}R_{1}+D_{i}^{-}R_{2}\right)\right\}+\delta^{2}\tilde{\Psi }\\ \psi_{12} & =\bar{B}V\\ \psi_{13} & =-\sum_{i=1}^{2^{m}}\chi_{i}\bar{B}\left(D_{i}R_{1}+D_{i}^{-}R_{2}\right)\\ \psi_{22} & =sym\left\{P_{2}W+R_{3}\bar{B}V\right\}+U_{\sigma }^{T}U_{\sigma }+\mu_{2}^{-2}I+\alpha_{2}I\\ \psi_{33} & =\mu^{2}\Psi^{-1}-2\mu Q_{1} \end{array}\right.$$

*and*

(44) $$\varepsilon <2\sqrt{\frac{\alpha_{1}\alpha_{2}\alpha_{3}}{\alpha_{1}+\alpha_{2}}}$$

*are solvable, and the adaptive regulation law of $\hat{M}\left(t\right)$ is designed by (14), the augmented system (21) will be stable. The gain matrices K, $H_{1}$, L and *Ψ* are, respectively, given by*

$$K=R_{1}Q_{1}^{-1},\;H_{1}=R_{2}Q_{1}^{-1},\;L=P_{2}^{-1}R_{3},\;\tilde{\Psi }=Q_{1}\Psi Q_{1}.$$

**Proof.**  Similar to Theorem 2, by taking the derivative of the functions given in (24) and (25), inequalities (26) and (27) can still be satisfied. As for the coupling term

$$e_{\sigma }^{T}\left(t\right)\sum_{i=1}^{2^{m}}\chi_{i}L\bar{B}\left(D_{i}K+D_{i}^{-}H_{1})\bar{x}(t_{k}\right)$$

in (34), we can conclude that, if the inequality (43) holds, then there must exist a parameter ε>0, depending on *L*, *K* and $H_{1}$, such that

(45) $$\begin{array}{c} e_{\sigma }^{T}\left(t\right)\sum_{i=1}^{2^{m}}\chi_{i}L\bar{B}\left(D_{i}K+D_{i}^{-}H_{1})\bar{x}(t_{k}\right)\leq \varepsilon ∥e_{\sigma }\left(t\right)∥(∥\bar{x}\left(t\right)∥+∥e_{k}\left(t\right)∥) \end{array}$$

By means of (43), (27) is translated as

(46) $$\begin{array}{cc} \dot{V_{2}} & \leq e_{\sigma }^{T}\left(t\right)\left(sym\left(P_{2}W\;+\;P_{2}L\bar{B}V\right)\;+\;P_{2}\bar{M}P_{2}\;+\;U_{\sigma }^{T}U_{\sigma }\right)e_{\sigma }\left(t\right)+\varepsilon ∥e_{\sigma }\left(t\right)∥\left(∥\bar{x}\left(t\right)∥+∥e_{k}\left(t\right)∥\right)\\ \; & +\sqrt{\frac{2\theta_{d}n_{1}}{\lambda_{min}\left(V^{T}V\right)}}∥P_{2}∥\left(\gamma \sqrt{n_{1}}P_{2}+\sqrt{tr(\bar{M})}\right)+2∥\hat{\sigma }\left(t\right)∥∥M^{*}{∥}_{F}^{2} \end{array}$$

Furthermore, by using (26) and (46), we can obtain

(47) $$\begin{array}{cc} \dot{V_{1}}+\dot{V_{2}} & \leq \xi^{T}\left(t\right)\Omega_{1}\xi \left(t\right)+\varepsilon ∥e_{\sigma }\left(t\right)∥∥\bar{x}\left(t\right)∥+\varepsilon ∥e_{\sigma }\left(t\right)∥∥e_{k}\left(t\right)∥+\mu_{1}^{2}y_{d}^{2}+\kappa \end{array}$$

where

$$\Omega_{1}=\left[\begin{array}{ccc} \varpi_{11} & P_{1}\bar{B}V & \varpi_{13}\\ {}* & \varpi_{22} & 0\\ {}* & * & -\Psi \end{array}\right].$$

By using the Schur lemma, we can attain

(43) ⟺ Ω_1_ < *diag*{*α*_1_*I*, *α*_2_*I*, *α*_3_*I*}

Then, (47) is inferred as

(48) $$\begin{array}{c} \dot{V_{1}}+\dot{V_{2}}=-{\bar{\xi }}^{T}\left(t\right)Y\bar{\xi }\left(t\right)+\mu_{1}^{2}y_{d}^{2}+\kappa \end{array}$$

where

(49) $$\begin{array}{c} \bar{\xi }\left(t\right)={\left[∥\bar{x}\left(t\right)∥,∥e_{\sigma }\left(t\right)∥,∥e_{k}\left(t\right)∥\right]}^{T},Y=\left[\begin{array}{ccc} \alpha_{1} & 0 & -\frac{\varepsilon }{2}\\ {}* & \alpha_{2} & -\frac{\varepsilon }{2}\\ {}* & * & \alpha_{3} \end{array}\right] \end{array}$$

It is noted that, if Y is a positive real matrix, the stability of system (21) can be pledged. Further, the characteristic polynomial of Y is described by

(50) $$\begin{array}{c} 4\alpha_{3}\lambda_{i}^{2}-4\left(\alpha_{1}\alpha_{3}+\alpha_{2}\alpha_{3}\right)\lambda_{i}+\left(4\alpha_{1}\alpha_{2}\alpha_{3}-\alpha_{1}\varepsilon^{2}-\alpha_{2}\varepsilon^{2}\right)=0 \end{array}$$

where $\lambda_{i}$ are the eigenvalues of Y. From (50), it is inferred that

$$\begin{array}{cc} \; & \lambda_{1}+\lambda_{2}=\alpha_{1}+\alpha_{2}>0\\ \; & \lambda_{1}\lambda_{2}=\frac{4\alpha_{1}\alpha_{2}\alpha_{3}-\left(\alpha_{1}+\alpha_{2}\right)\varepsilon^{2}}{4\alpha_{3}} \end{array}$$

If the condition (44) is met, then it is easy to conclude that $\lambda_{1}\lambda_{2}>0$. To sum up, the matrix Y is a positive real matrix and thus the augmented system (21) is proved to be stable. □

## 6. Simulation

Consider the A4D aircraft model as the controlled system. In a flight environment of 16,000 ft altitude and 0.9 Mach, the dynamics of the A4D system can be modeled by (1), where $x\left(t\right)\in R^{4}$ represents the state of the aircraft, $x_{1}\left(t\right)$ is the forward velocity $\left(ft\cdot s^{-1}\right)$, $x_{2}\left(t\right)$ is the attack angle (rad), and $x_{3}\left(t\right)$ and $x_{4}\left(t\right)$ are the velocity of pitch $\left(rad\cdot s^{-1}\right)$ and the angle of pitch (rad), respectively. u(t) is the elevator deflection (deg) and the output z(t) is selected as the forward velocity $x_{1}\left(t\right)$. Similar to [45,46], the dynamic model was modeled by using the principle of system identification. Based on the idea of sparse identification, the input and output data of A4D aircraft were identified by the generalized least squares method, and then the state parameter matrices *A*, *B* and *C* of the system were obtained as

$$A=\left[\begin{array}{cccc} -0.0605 & 32.38 & 0 & 32\\ -0.0015 & -1.47 & 1 & 0\\ -0.0111 & -34.72 & -2.793 & 0\\ 0 & 0 & 1 & 0 \end{array}\right],B=\left[\begin{array}{c} 0\\ -0.1064\\ -33.8\\ 0 \end{array}\right],C=\left[\begin{array}{cccc} 1 & 0 & 0 & 0 \end{array}\right]$$

Next, we considered the anti-disturbance control for two types of irregular disturbances by selecting different excitation functions.

First, in order to describe attenuated harmonic (AH) disturbances, the DNN parameters of the disturbance model were selected as

$$W=\left[\begin{array}{cc} 0 & 4\\ -4 & 0 \end{array}\right],\;\;V=\left[\begin{array}{cc} 0.7 & 0 \end{array}\right],\;\;M^{*}=\left[\begin{array}{cc} -0.3 & -0.05\\ 0.01 & 0.45 \end{array}\right],\;\;\Phi \left(t\right)=\left[\begin{array}{c} \arctan(t)\\ \arctan(t) \end{array}\right]$$

Preselect the candidate value of $R_{3}$ as

$$R_{3}=\left[\begin{array}{ccccc} 0 & -30.3493 & -5.6140 & 0 & 0\\ 0 & 50.1378 & 10.9620 & 0 & 0 \end{array}\right].$$

Meanwhile, by defining $\mu_{1}=\mu_{2}=1$ and solving inequalities (23), (34), (35), we obtained

$$K=\left[\begin{array}{ccccc} 0.0038 & -0.6781 & 0.0339 & 0.7744 & 0.0008 \end{array}\right]$$

$$L=10^{-6}*\left[\begin{array}{ccccc} 0 & -0.1055 & -0.0195 & 0 & 0\\ 0 & 0.1743 & 0.0381 & 0 & 0 \end{array}\right]$$

$$H_{1}=\left[\begin{array}{ccccc} 0.0014 & -0.1912 & 0.0115 & 0.233 & 0.0005 \end{array}\right]$$

Assume that the initial conditions of the augmented states and the desired output are selected as

$$x_{0}={\left[2,-2,3,-2\right]}^{T},\;\;\sigma_{0}={\left[4,4\right]}^{T},\;\;z_{d}=18$$

**Figure 1:**
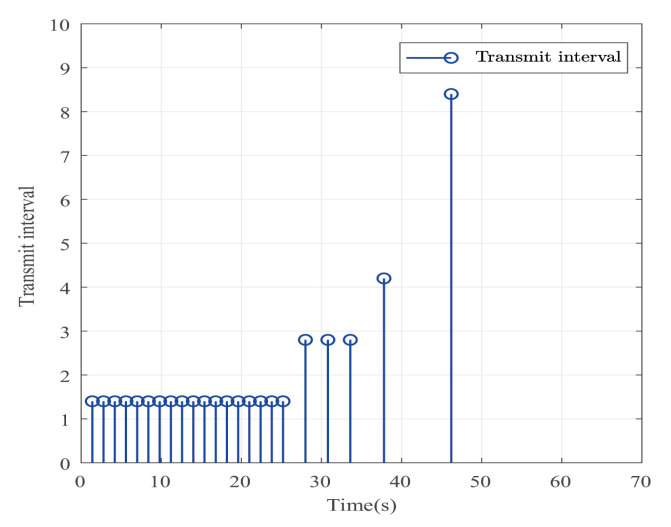
The event-triggered release times and intervals in the case of AH disturbances.

**Figure 2:**
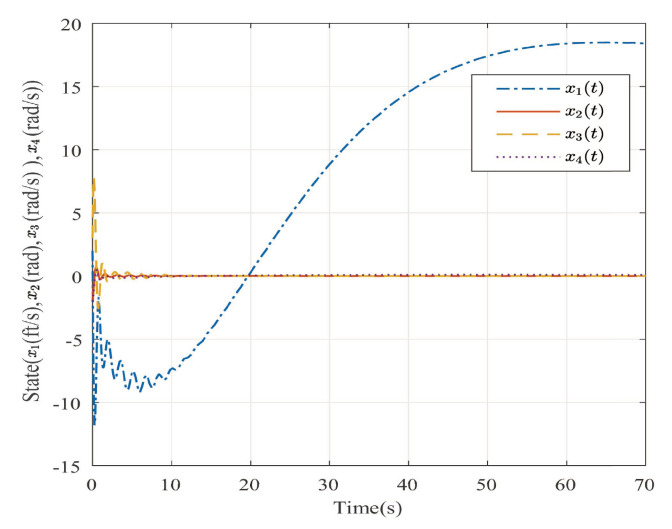
The trajectories of the system states in the case of AH disturbances.

**Figure 3:**
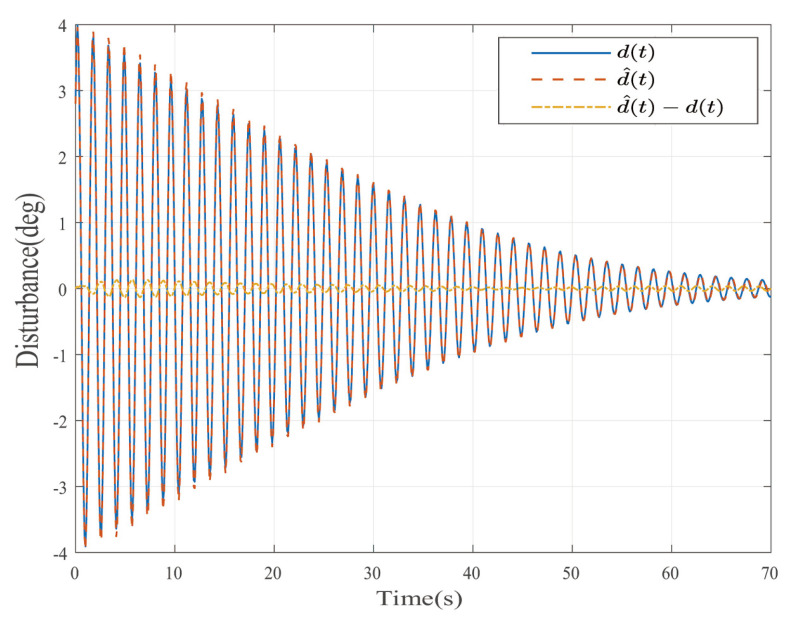
The disturbance estimates and estimation error in the case of AH disturbances.

**Figure 4:**
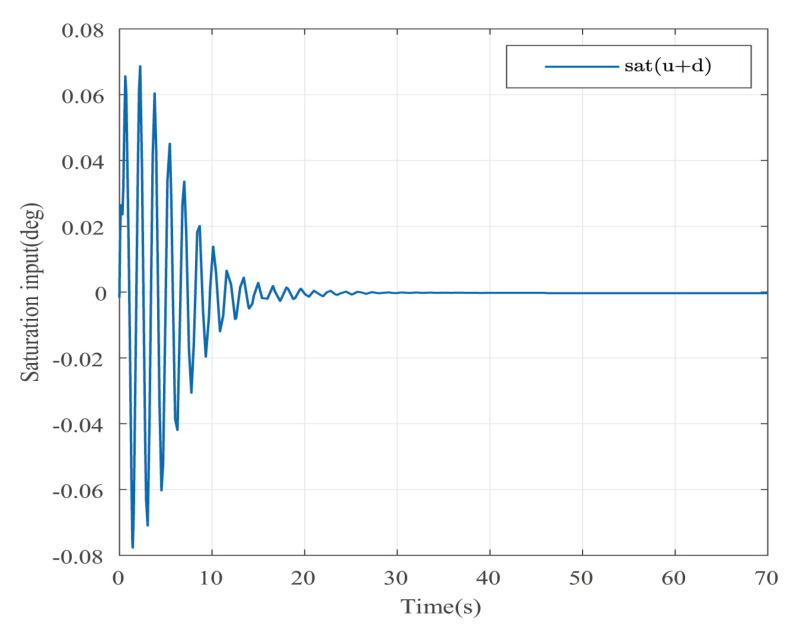
The dynamics of the saturated control input in the case of AH disturbances.

**Figure 5:**
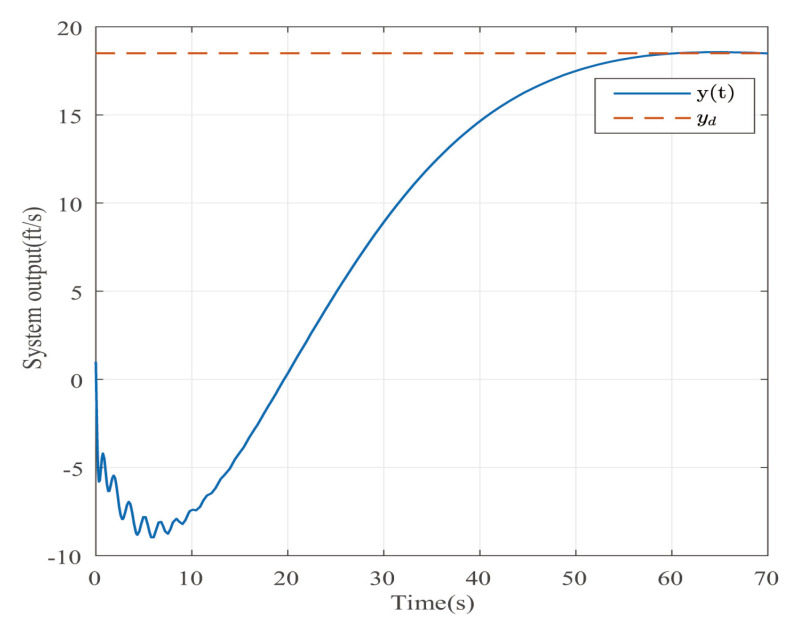
The trajectory of the system output in the case of AH disturbances.

**Figure 6:**
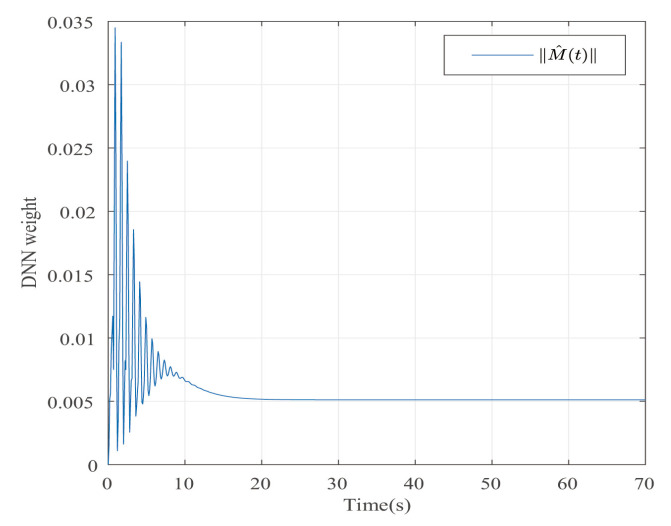
The trajectory of the dynamical weights in the case of AH disturbances.

Suppose that Ψ is an identity matrix, δ=0.01. Figure 1 reflects the triggered release time and the corresponding interval. The dynamics of the states are plotted in Figure 2, which can reflect the favorable stability. Both the attenuated harmonic disturbances and the disturbance estimated value together with the estimated error are displayed in Figure 3. Thus, the satisfactory capacities of disturbance modeling and estimation are fully embodied. Figure 4 and Figure 5 depict the dynamical trajectories of the input and output, respectively, which verifies the favorable input constraint and dynamical tracking performance. The dynamics of the DNN weight are exhibited in Figure 6.

Second, sawtooth wave (STW) signals usually appear in some circuit or electromagnetism systems, and it is quite hard to monitor them using common epitaxial systems. For modeling STW disturbances, the specific parameters of DNNs are considered as

$$W=\left[\begin{array}{cc} 0 & -6\\ 2 & -0.01 \end{array}\right],\;\;V=\left[\begin{array}{cc} -0.01 & -1 \end{array}\right],\;\;M^{*}=\left[\begin{array}{cc} 0 & 0.02\\ -0.2 & 0.45 \end{array}\right]$$

$$\Phi \left(t\right)=\left\{\;\;\;\begin{array}{cc} \left[\begin{array}{c} \frac{1}{1+e^{-0.5t}}\\ \frac{1}{1+e^{-0.5t}} \end{array}\right] & t\geq 0\;\\ \left[\begin{array}{c} -2.1\\ -2.1 \end{array}\right] & t<0 \end{array}\right.$$

By solving inequalities (23), (34) and (35), the gains *K*, *L* and $H_{1}$ can be found to be

$$K=\left[\begin{array}{ccccc} 0.0231 & 0.2521 & 0.0184 & 0.9451 & 0.0431 \end{array}\right]$$

$$L=10^{-6}*\left[\begin{array}{ccccc} 0 & -0.1441 & -0.0266 & 0 & 0\\ 0 & 0.0792 & 0.0173 & 0 & 0 \end{array}\right]$$

$$H_{1}=\left[\begin{array}{ccccc} -0.1475 & 0.0062 & 0.0230 & 0.1648 & 0.0430 \end{array}\right]$$

Suppose that the initial values are, respectively, given by

$$x_{0}={\left[2,-2,3,-2\right]}^{T},\;\;\sigma_{0}={\left[3,3\right]}^{T}.$$

**Figure 7:**
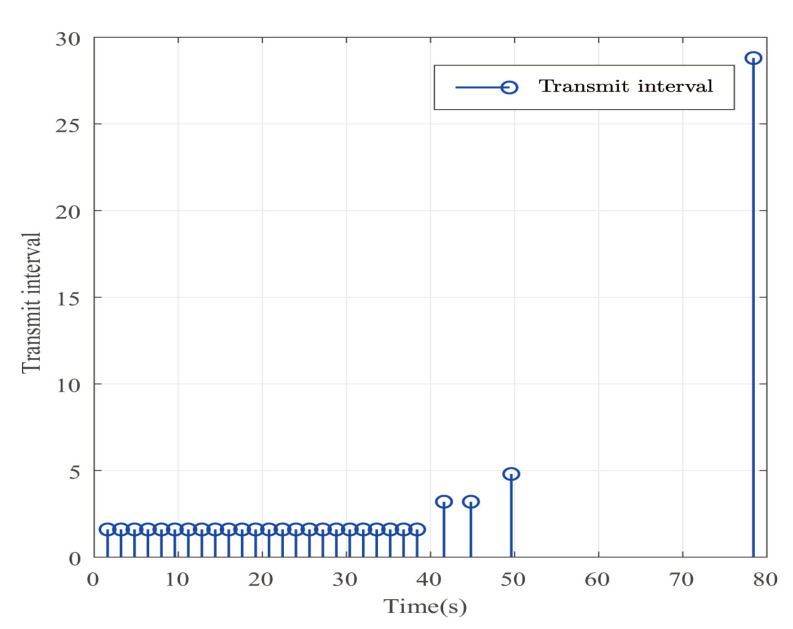
The event-triggered release times and intervals in the case of STW disturbances.

**Figure 8:**
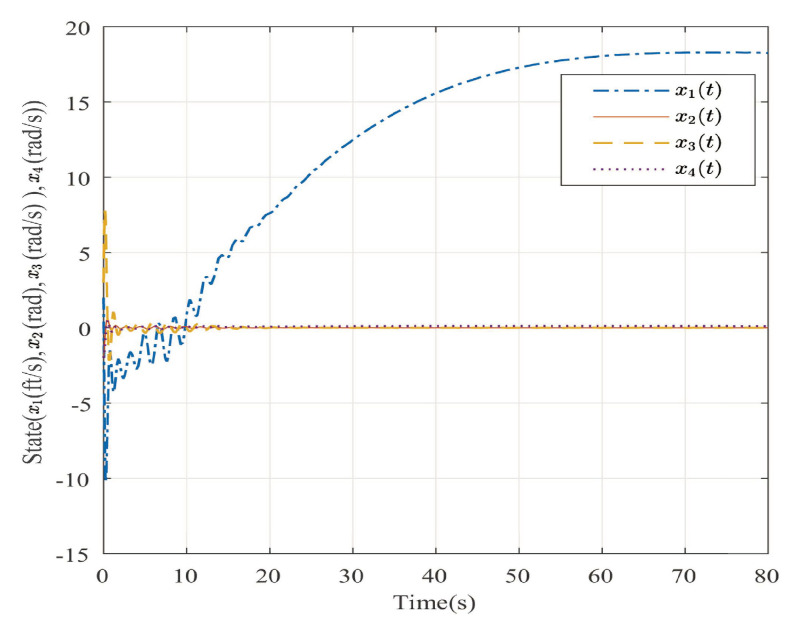
The trajectories of the system states in the case of STW disturbances.

**Figure 9:**
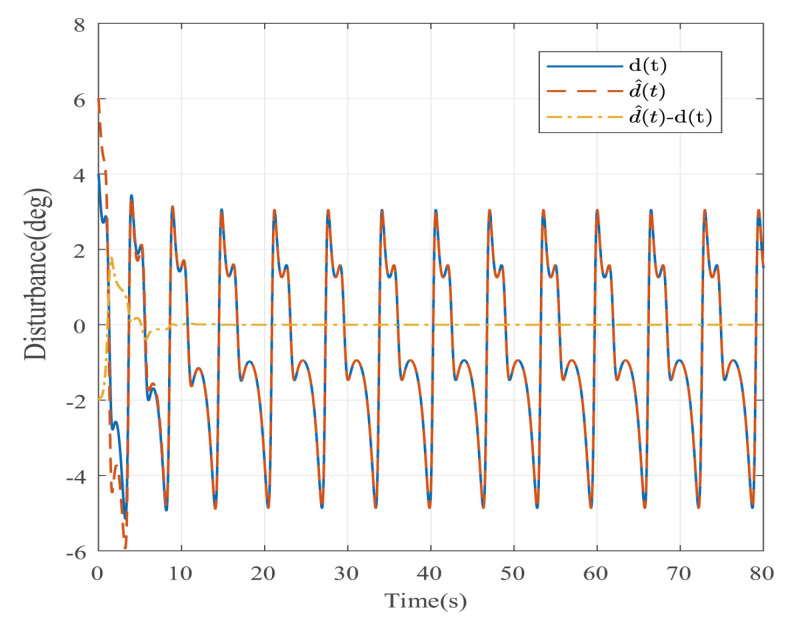
The disturbance estimates and estimation error in the case of STW disturbances.

**Figure 10:**
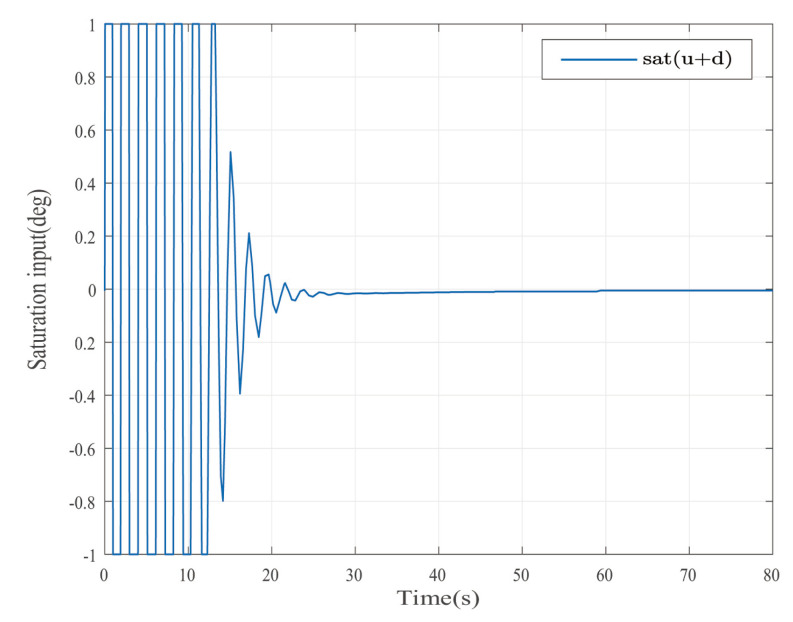
The dynamics of the saturated control input in the case of STW disturbances.

**Figure 11:**
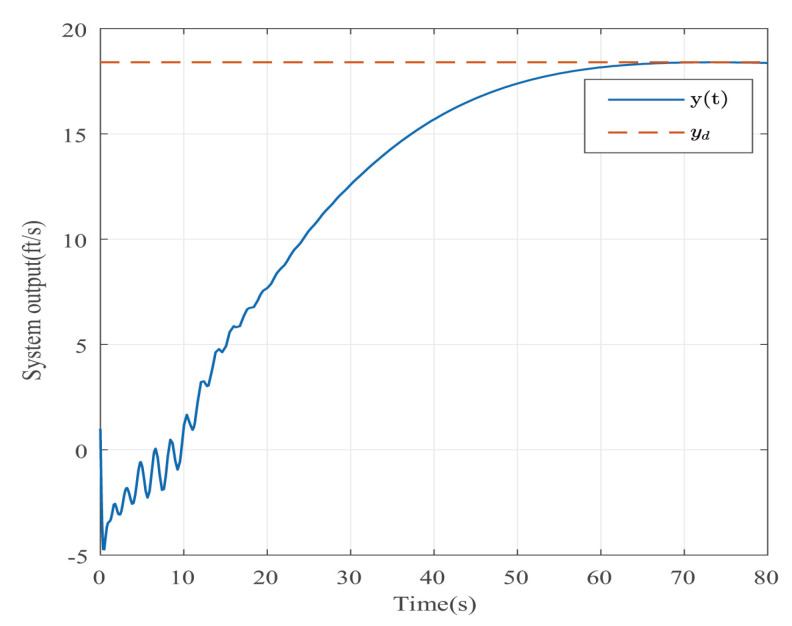
The trajectory of the system output in the case of STW disturbances.

**Figure 12:**
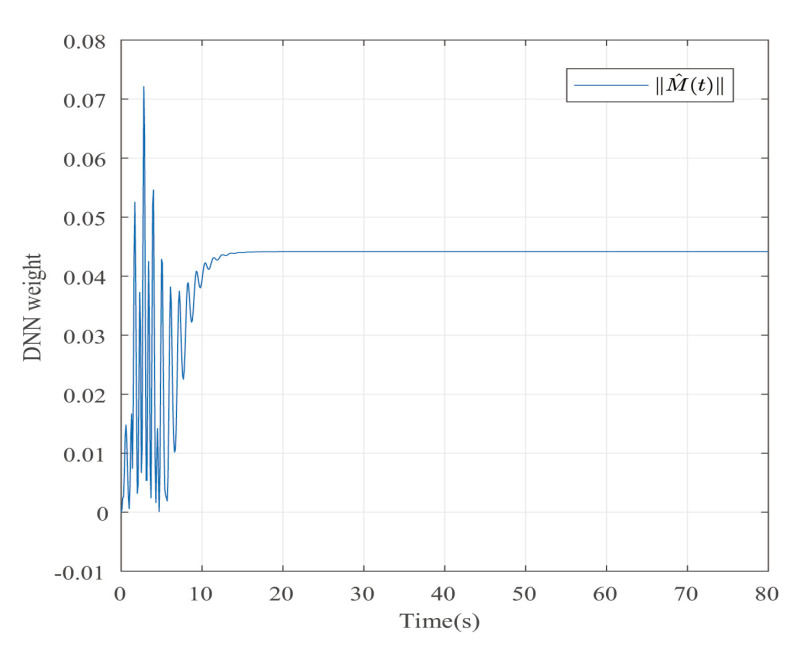
The trajectory of the dynamical weights in the case of STW disturbances.

The desired output is defined as $z_{d}=17$. The triggered release time and corresponding intervals are displayed in Figure 7. Figure 8 is the tracks of the states of the A4D system. Figure 9 exhibits the dynamics of STW and its estimates. Figure 10 and Figure 11, respectively, present the saturated input and the system output. Figure 12 depicts the dynamics of the designed DNN weight. Figure 8, Figure 9, Figure 10, Figure 11 and Figure 12 demonstrate that the designed event-triggered PI control input can obtain favorable control performances in the case of STW disturbances while saving a considerable amount of resources (see Figure 7).

By effectively estimating for AH and STW disturbances, respectively, a satisfactory anti-disturbance control frame can be embodied in the above simulation. Compared to those results that rely on constant or harmonic disturbances, the main advantages of the suggested method are reflected in wider anti-disturbance ranges, more objective control tasks and less data transfer. Of course, some existing disadvantages—for example, more conservative algorithms and higher real-time requirements—need to be fully considered in the future work.

## 7. Conclusions

In this paper, a valid anti-disturbance event-triggered control probelm is discussed for systems with multiple constraints under the frame of DNN disturbance modeling. Different from the usual time-triggered problem, the whole algorithm design was made with the event-triggered frame. After constructing the augmented event-triggering condition, a novel event-triggered DOBAC algorithm was designed by integrating the modified adaptive regulation law with the DNN disturbance models. Meanwhile, a composite event-triggered controller was successively designed with a polytopic description of the saturated actuator. By using the convex optimization theory, the relevant proofs were given to verify the stability of the closed-loop augmented system and to meet the multiple constraints regarding the augmented states, as well as the system output. Moreover, the dynamics of the tracking error can be displayed as converging to zero. Finally, the simulation results illustrate that the proposed scheme is effective in terms of desired control performances and significantly reduced resource utilization.

## Data Availability

Not applicable.

## References

[1] Chen W.H., Ballanceand D.J., Gawthrop P.J. (2000). A Nonlinear Disturbance Observer for Robotic Manipulators. IEEE Trans. Ind. Electron..

[2] Zhang H.F., Wei X.J., Karimi H.R., Han J. (2017). Anti-Disturbance Control Based on Disturbance Observer for Nonlinear Systems with Bounded Disturbances. J. Frankl. Inst..

[3] Nguyen M.H., Dao H.V., Ahn K.K. (2022). Adaptive Robust Position Control of Electro-Hydraulic Servo Systems with Large Uncertainties and Disturbances. Appl. Sci..

[4] Abdul-Adheem W.R., Alkhayyat A., Al Mhdawi A.K., Bessis N., Ibraheem I.K., Abdulkareem A.I., Humaidi A.J., AL-Qassar A.A. (2021). Anti-Disturbance Compensation-Based Nonlinear Control for a Class of MIMO Uncertain Nonlinear Systems. Entropy.

[5] Zhou L., Tse K.T., Hu G., Li Y. (2021). Higher Order Dynamic Mode Decomposition of Wind Pressures on Square Buildings. J. Wind. Eng. Ind. Aerodyn..

[6] Nguyen M.H., Dao H.V., Ahn K.K. (2022). Extended Sliding Mode Observer-Based High-Accuracy Motion Control for Uncertain Electro-Hydraulic Systems. Int. J. Robust Nonlinear Control.

[7] Zong G.D., Qi W.H., Karimi H.R. (2021). *L*_1_ Control of Positive Semi-Markov Jump Systems with State Delay. IEEE Trans. Syst. Man Cybern. Syst..

[8] Zhong Z.X., Wang X.Y., Lam H.K. (2021). Finite-Time Fuzzy Sliding Mode Control for Nonlinear Descriptor Systems. IEEE/CAA J. Autom. Sin..

[9] Gao Z. (2016). Active Disturbance Rejection Control for Nonlinear Fractional Order Systems. Int. J. Robust Nonlinear Control.

[10] Chen P., Luo Y., Peng Y., Chen Y. (2021). Optimal Fractional-Order Active Disturbance Rejection Controller Design for PMSM Speed Servo System. Entropy.

[11] Aishwarya A., Ujjwala T., Vrunda J. (2019). Disturbance Observer Based Speed Control of PMSM Using Fractional Order PI Controller. IEEE/CAA J. Autom. Sin..

[12] Hua Z.G., Chen M. (2022). Coordinated Disturbance Observer-Based Flight Control of Fixed-Wing UAV. IEEE Trans. Circuits Syst. II Exp. Briefs.

[13] Zhang J.H., Zheng W.X., Xu H., Xia Y.Q. (2021). Observer-Based Event-Driven Control for Discrete-Time Systems with Disturbance Rejection. IEEE Trans. Cybern..

[14] Li R., Zhu Q., Yang J., Narayan P., Yue X. (2021). Disturbance-Observer-Based U-Control (DOBUC) for Nonlinear Dynamic Systems. Entropy.

[15] Wang X.Y., Li S.H., Wang G.D. (2020). Distributed Optimization for Disturbed Second-Order Multi-Agent Systems Based on Active Anti-Disturbance Control. IEEE Trans. Neural Netw. Learn. Syst..

[16] Yi Y., Zheng W.X., Sun C.Y., Guo L. (2016). DOB Fuzzy Controller Design for Non-Gaussian Stochastic Distribution Systems Using Two-Step Fuzzy Identification. IEEE Trans. Fuzzy Syst..

[17] Zhao Z.J., Ahn C.K., Li H.X. (2020). Boundary Anti-Disturbance Control of a Spatially Nonlinear Flexible String System. IEEE Trans. Ind. Electron..

[18] Hu T.S., Lin Z. (2001). Control Systems with Actuator Saturation: Analysis and Design.

[19] Tarbouriech S., Garcia G., Gomes J.M., Queinnec I. (2011). Stability and Stabilization of Linear Systems With Saturating Actuators.

[20] Fridman E., Pila A., Shaked U. (2003). Regional Stabilization and *H*_∞_ Control of Time-Delay Systems with Saturating Actuators. Int. J. Robust Nonlinear Control.

[21] Zhou B., Zheng W.X., Duan G.R. (2011). An Improved Treatment of Saturation Nonlinearity with Its Application to Control of Systems Subject to Nested Saturation. Automatica.

[22] Wei Y.L., Zheng W.X., Xu S.Y. (2015). Anti-Disturbance Control for Nonlinear Systems Subject to Input Saturation via Disturbance Observer. Syst. Control Lett..

[23] Li Y.L., Lin Z.L. (2015). A Complete Characterization of the Maximal Contractively Invariant Ellipsoids of Linear Systems Under Saturated Linear Feedback. IEEE Trans. Autom. Control.

[24] Bai W.W., Zhou Q., Li T.S., Li H.Y. (2020). Adaptive Reinforcement Learning Neural Network Control for Uncertain Nonlinear System with Input Saturation. IEEE Trans. Cybern..

[25] Wang X.L., Ding D.R., Dong H.L., Zhang X.M. (2021). Neural-Network-Based Control for Discrete-Time Nonlinear Systems with Input Saturation Under Stochastic Communication Protocol. IEEE/CAA J. Autom. Sin..

[26] Pan H.H., Sun W.C., Gao H.J., Jing X.J. (2016). Disturbance Observer-Based Adaptive Tracking Control with Actuator Saturation and Its Application. IEEE Trans. Autom. Sci. Eng..

[27] Li Z.J., Zhao J. (2021). Adaptive Consensus of Non-Strict Feedback Witched Multi-Agent Systems with Input Saturations. IEEE/CAA J. Autom. Sin..

[28] Tee K.P., Ren B.B., Ge S.S. (2011). Control of Nonlinear Systems with Time Varying Output Constraints. Automatica.

[29] Ngo K.B., Mahony R., Jiang Z.P. Integrator Backstepping Using Barrier Functions for Systems with Multiple State Constraints. Proceedings of the 44th IEEE Conference on Decision and Control.

[30] Meng W.C., Yang Q.M., Si S.N., Sun Y.X. (2016). Adaptive Neural Control of a Class of Output-Constrained Non-Affine Systems. IEEE Trans. Cybern..

[31] Liu Y.J., Ma L., Liu L., Tong S.C., Chen C.L.P. (2020). Adaptive Neural Network Learning Controller Design for a Alass of Nonlinear Systems with Time-Varying State Constraints. IEEE Trans. Neural Netw. Learn. Syst..

[32] Astrom K., Bernhardsson B. Comparison of Periodic and Event Based Sampling for First-Order Stochastic Systems. Proceedings of the 14th IFAC World Congress.

[33] Sahoo A., Xu H., Jagannathan S. (2016). Neural Network-Based Event Triggered State Feedback Control of Nonlinear Continuous-Time Systems. IEEE Trans. Neural Netw. Learn. Syst..

[34] Dolk V., Borgers D., Heemels W.P.M.H. (2017). Output-Based and Decentralized Dynamic Event-Triggered Control with Guaranteed *L*_p_-Gain Performance and Zeno-Freeness. IEEE Trans. Autom. Control.

[35] Chen P., Li F.Q. (2018). A Survey on Recent Advances in Event-Triggered Communication and Control. Inf. Sci..

[36] Wang W., Li Y.M., Tong S.C. (2021). Neural-Network-Based Adaptive Event-Triggered Consensus Control of Nonstrict-Feedback Nonlinear Systems. IEEE Trans. Neural Netw. Learn. Syst..

[37] Li Y.X., Yang G.H. (2018). Model-Based Adaptive Event-Triggered Control of Strict-Feedback Nonlinear Systems. IEEE Trans. Neural Netw. Learn. Syst..

[38] Wu Z.G., Xu Y., Pan Y.J., Su S.H., Tang Y. (2018). Event-Triggered Control for Consensus Problem in Multi-Agent Systems with Quantized Relative State Measurements and External Disturbance. IEEE Trans. Circuits Syst. I Reg. Pap..

[39] Zhang Y.H., Sun J., Liang H.J., Li H.Y. (2020). Event-Triggered Adaptive Tracking Control for Multiagent Systems with Unknown Disturbances. IEEE Trans. Cyber..

[40] Ren C.E., Fu Q.X., Zhang J.G., Zhao J.S. (2021). · Adaptive Event-triggered Control for Nonlinear Multi-agent Systems with Unknown Control Directions and Actuator Failures. Nonlinear Dyn..

[41] Yang L.W., Liu T., Hill D.J. (2021). Decentralized Event-Triggered Frequency Control with Guaranteed *L*_∞_-Gain for Multi-Area Power Systems. IEEE Control Syst. Lett..

[42] Deng Y.J., Zhang X.K., Im N.K., Zhang G.Q., Zhang Q. (2020). Model-Based Event-Triggered Tracking Control of Underactuated Surface Vessels with Minimum Learning Parameters. IEEE Trans. Neural Netw. Learn. Syst..

[43] Yu W., Rosen J. (2013). Neural PID Control of Robot Manipulators with Application to an Upper Limb Exoskeleton. IEEE Trans. Cybern..

[44] Han H.G., Zhang L., Hou Y., Qiao J.F. (2016). Nonlinear Model Predictive Control Based on a Self-Organizing Recurrent Neural Network. IEEE Trans. Neural Netw. Learn. Syst..

[45] Guo L., Chen W.H. (2005). Disturbance Attenuation and Rejection for Systems with Nonlinearity via DOBC Approach. Int. J. Robust Nonlinear Control.

[46] McRuer D., Ashkenas I., Graham D. (1976). Aircraft Dynamics and Automatic Control.

